# Reversible and Irreversible Processes in Drying and Wetting of Soil

**DOI:** 10.3390/ma13010135

**Published:** 2019-12-28

**Authors:** Ilie Bodale, Alexandru Stancu

**Affiliations:** 1 Faculty of Horticulture, University of Agricultural Sciences and Veterinary Medicine, 3 Mihail Sadoveanu Alley, 700490 Iasi, Romania; ilbodale@gmail.com; 2 Faculty of Physics, Alexandru Ioan Cuza University of Iasi, 11 Boulevard Carol I, 700506 Iasi, Romania

**Keywords:** soil–water characterization curve (SWCC), FORC diagram, Preisach model, reversible and irreversible processes, wetting–drying processes

## Abstract

In this article, we provide a detailed description of a modeling technique for the capillary hysteresis in a soil-like porous material based on a Generalized Preisach Model. The identification of the reversible and irreversible Preisach distributions was performed with the first-order reversal curve (FORC) diagram technique, which is very popular now in magnetism and in other areas of science to give a fingerprint of the studied system. A special attention was given to the evaluation of the reversible component. In this case, we used a set of data published in 1965 by Morrow and Harris which has been used as a reference by many other researchers since. The advantage of this approach is that the experimental FORC distributions can be described with analytical functions and easily implemented in the mentioned Preisach-type model. Our research is also focused on the development of a characterization tool for the soil using the soil-moisture hysteresis. The systematic use of scanning curves provides a (FORC) diagram linked to the physical properties of the studied soil. The agreement between the experimental data and the Preisach model using the set of parameters found through the FORC technique is really noticeable and gives a good practical option to the researchers to use a method with a strong predictive capability.

## 1. Introduction

The proportion of water from soil is usually estimated by using the soil–water characterization curve (SWCC) defined as the relationship between matric suction and the water content [1]. The matric suction represents the difference between pore-air and pore-water pressures which offer information about the amount of water and the energy state in liquid phase.

The matric suction of soil presents a memory effect in unsaturated states [2]. This memory effect is essentially evidenced by the hysteretic behavior during the wetting and drying processes. The profound understanding of this complex physical process is essential for a variety of scientific fields like the soil physics [3], characterization methods of pore size distribution in mesoporous materials [4], the science of wetting with applications in many exciting modern industrial fields like cosmetics, pharmaceutical, oil recovery, lubrication, liquid coating nanofluidics and electrowetting, and many other areas [5].

In soil, the hysteretic behavior is determined by a number of factors. Among the most important are the irregular pore space geometry, different contact angle at the two directions of evolution of the liquid content, the entrapped air inside the media, shrinking and swelling of pores—but also by the thermal effect [6,7,8,9,10]. The vertical circulation of water in the pore is characterized by ‘ink bottle effect’, described as the effect given by geometric non-uniformity of individual pores when the water passes by smaller cross-sections of pores. The contact angle for the advancing (wetting of pore) and receding (drying of pore) interface have different values. The advancing angle is larger than the receding one, thus the suction at drying is greater than wetting. When the liquid phase is dominant in pores, the air trapped inside slows the filling pore process. In fine grained media, the swelling of the pore at wetting and shrinkage at drying changes the diameters of the pores. Additionally, the variation of temperature affects the fluid retention through the change in the fluid viscosity and density.

In the last five decades, a number of models have been developed to estimate the water retention in porous media which is a really important step in the profound understanding of wetting/drying processes. The models in this domain are usually developed starting from well-known facts, like the mass conservation of water law, Jurin’s law for the level of a liquid within a capillary tube, and the flow of water through a porous medium according to Darcy’s law [11]. The models are based on the following hypotheses: the pore behavior is not influenced significantly by the surrounding pores (independent pores) [12] and the pores are connected through small tubes creating a network of pores (dependent pores) [13]. The dependent domain method assumes a correlation between the wetting and drying processes of all the pores in porous media.

In the early stages of the development of the models of hysteresis, the main inspiration source was the article published in 1935 by Ferenc Preisach [14] for ferromagnetic hysteresis. In this article, Preisach is introducing the concept of rectangular hysteron as the elementary element of hysteresis. Actually, this idea can be found in an incipient form even in the articles published by Weiss and de Freudenreich in 1916 [15]. The ferromagnetic sample was represented in the Preisach model by a distribution of hysterons which is now called the Preisach distribution. In the 1940s and 1950s, a number of researchers have developed and analyzed further the properties of the “domain model” with both dependent and independent domains versions [16,17]. Poulovassilis, based on this evolution, has developed in 1962 a model for the hysteresis of pore water using independent (non-interacting) “domains” [18], on which Philip [19] and Mualem [20,21,22,23] have further improved in a number of models. In all these papers, the main goal was to identify the model’s parameters using sets of experimental data. Identification techniques become more sophisticated and the results have shown improved fits of the data available.

A number of physical models were developed to evaluate the soil–water retention in various conditions (van Genuchten [24], Zhou et al. [25], Chen et al. [26]). This essential parameter can be estimated within a few percent’s error in well controlled structures and it was tested with experimental data. However, to model an entire system of scanning curves within the major hysteresis curve is a much more complex problem (Pham [27]) in which a specialized hysteresis model has to be used.

However, it is only recently that a significant effort was invested in the systematic use of the Preisach model, taking into account the mathematical properties of this model as they were evidenced in the works of Krasnosel’skii and Pokrovskii [28]. For a very good review of this modeling approach, we recommend Chapter 7 from the excellent book “The Science of Hysteresis” (editors: Giorgio Bertotti and Isaak Mayergoyz) [29]. In this chapter named “Application of the Preisach Model to Soil-Moisture Hysteresis,” the authors are introducing the Preisach model and show how this classical hysteresis model can be applied for the soil-moisture hysteresis. More details on their modeling technique can be found in [30,31,32,33].

**Figure 1:**
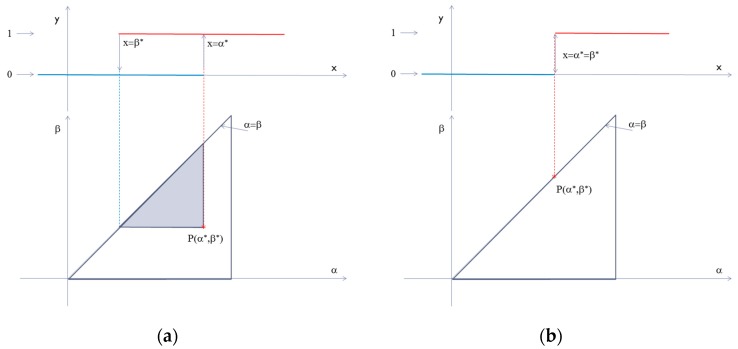
(**a**) Irreversible rectangular hysteron associated to one point in the Preisach plane; (**b**) reversible rectangular hysteron associated to one point in the Preisach plane.

Essentially, as in any Preisach-type model, we base the soil-moisture hysteresis on a distribution of rectangular hysterons characterized by the two values of the input parameter, at which the switches between the two states occur (0 and 1, which could be understood as “empty” and “filled” states of a physical structure in the porous medium)—see Figure 1a. If the switches between the two limits take place at two different values for the input parameter, then one can consider this fundamental brick in the model as an irreversible element of hysteresis. The entire system of such fundamental entities is profoundly linked with the observed hysteretic behavior in successive wetting/drying processes and with energy flow between the system and the environment. The width of the hysterons may be linked to the shape of the real physical sub-structures in the soil. In order to take into account also physical sub-structures with non-hysteretic behavior in the Preisach model, one can add degenerated hysterons with zero width (see Figure 1b). These hysterons are allocated to the first bisector of the Preisach plane and they represent completely reversible processes. In a complex process in which the input parameter has a certain variation, the final value of the output is calculated as the sum of the all hysterons in the “filled” state. In the Preisach model, one can calculate the separation line between the hysterons in the “empty” and “filled” states. This shows the importance of evaluation of the entire distribution of the hysterons as a function of their positions in the Preisach plane. This process is named identification and is the fundamental element in the Preisach characterization of any system with hysteresis. The mentioned distribution, also called Preisach distribution, is in principle uniquely linked to a studied sample. In many studies of systems with hysteresis, the systematic evaluation of the Preisach distributions have shown that they have specific features linked to the physical structure of the system. One can expect the same behavior in the case of Preisach modeling of various categories of porous materials, including soils. With this interpretation in mind, one can look at the identification of the Preisach distribution as a characterization tool for the physical structure of the studied system.

**Figure 2:**
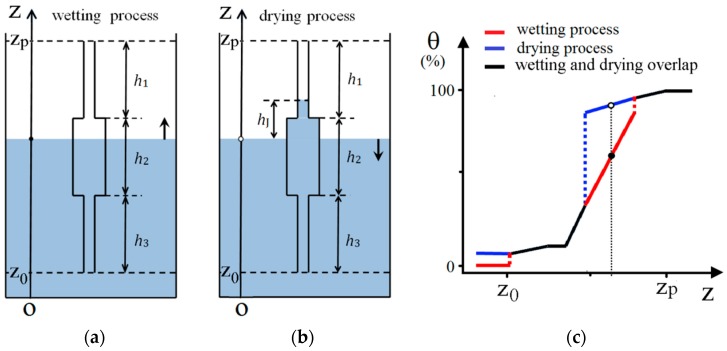
Independent pore in (**a**) wetting and (**b**) drying processes. The hysteresis behavior of the independent pore (**c**) with a tank in the middle and capillary tubes at the ends.

To understand the specificity of the soil-moisture hysteresis, we shall start the discussions on the Preisach density function from the shape of the hysteresis loops of the constitutive elements of the actual physical system and, after that analysis, we shall present an identification technique for the real system based on the use of experimental first-order reversal curves. To make this approach as simple as possible, we take into account a possible elementary brick for the hysteretic behavior in the wetting (Figure 2a) and drying process (Figure 2b) of a porous material of a cylinder-shaped pore. The geometrical data for this pore are the lengths of the three regions with different sections. In our hypothesis, we took the radius of the sections one as the same size as the three, and different radius for the second region which is larger. We considered that the capillary effects are evident only in the regions one and three which obeys Jurin’s law with the characteristic height *h_J_*. The capillarity effect was neglected in the second region where the radius of the pore is larger than a capillary tube.

The level of the liquid in the exterior is the independent variable (*z*) and the percentage of the volume of the pore filled with the liquid. The drying process is started when the liquid in the exterior is at the level *z = z_p_* and the pore is filled 100%. After the entire pore is emptied (*z = z*_0_ corresponding to 0%) and the level of the liquid increases, then the wetting process occurs. In fact, in field it is very difficult to remove all water from the soil as this can be done only in the laboratory. In Figure 2c, we show the dependence of the filling percentage of the pores (named usually θ) as a function of *z* on both drying and wetting processes which show a hysteretic behavior. From the theoretical point of view, it is important to say that the shape of this hysteresis loop can be controlled by the geometrical parameters characterizing the pore. Additionally, an important fact that we have to mention is that the hysteresis loop is far from having a rectangular shape, as it is required in the Classical Preisach Model (CPM). Essentially, this means that we have to add to the model a reversible component, which can account for the variations in the measured filling degree which are not due to irreversible processes. A simple analysis for the pore shape we have considered shows that the reversible component is dependent on the radii of the cylinders in the three distinct regions.

In this article, we present a characterization technique for the soil-moisture hysteresis using a Preisach-type model which includes both reversible and irreversible hysteretic components. A set of first-order reversal curves (FORC) [34], also known as primary scanning curves (e.g., re-drying and re-wetting curves) [35] are used to find an appropriate mathematical fitting function that can be used as a Preisach distribution in the model. The FORC diagram technique is presented in the third section of the article. The results of the identification performed on a set of experimental data from the literature are also provided and discussed in the final part of the article.

## 2. Classical and Generalized Preisach Models

The Classical Preisach Model (CPM) was, for many years, used to describe a wide range of hysteretic processes observed in physics but also in other fields like biology or economics [36,37,38]. There is still a strong debate between the researchers using this model if the hysteron has or should have a link with a physical entity in the sample under examination. Some researchers, especially the mathematicians, are advocating the idea of a clear separation between the physical hysteretic process and the model. In their view, CPM is nothing more than a purely mathematical way to interpolate complex sets of data showing hysteresis behavior. At the other limit are the researchers placing a lot of emphasis in the existence of a physical foundation for the model in the existence of hysteretic entities that can be seen as the fundamental bricks for the model at the microscopic level. Our opinion is that if we use the model only as a mathematical tool, we are losing a central element in any modeling attempt. In this case, we lose the ability to give an insight about the physical system from the experimental data. In magnetism, where the model originated, the same opinions are present but in recent years, we have seen a strong interest in the idea of finding essential physical elements from the experimental investigation of various magnetic systems. This trend could be related to the identification technique mentioned in the introduction, the FORC diagram method. In magnetism, the FORC distributions obtained from experimental data are seen as powerful tools to evidence the distributions of coercive and interaction fields characteristic to the studied systems [39]. Starting from this idea, a lot of research effort was invested to find a link between the local microscopic magnetic entities showing hysteresis and the distributions evidenced by the FORC technique. In this article, we propose to use a similar approach, starting with the evaluation of the hysteresis behavior on a single pore (hysteron), followed by the use of the FORC technique to analyze what kind of physical data can be obtained.

When applied to the drying/wetting process of a set of independent pores, we observe that there is no easy way to define the two states that are specific to a rectangular hysteron. Usually, in soil, the two states considered are the filled with water and empty pore, however, looking at the one pore hysteresis we see that this is not really true. For some parameters, we can come closer to the “ideal” hysteron, but with other parameters we actually do not. Another aspect that becomes obvious is that the drying/wetting hysteron shows the reversible part, as mentioned before. Moreover, the symmetry we have usually in the ferromagnetic hysteresis is not present here, and the reversible susceptibilities on the upper and lower branches of the hysteresis loop are not equal. To model a system of rectangular hysterons with these characteristics, we can use a version of CPM that includes also a singular distribution of hysterons with zero coercivity, named the Generalized Preisach Model (GPM) [40] associated with the distribution of reversible processes. It is important to mention that GPM is a straightforward extension of the CPM and that it is still conditioned by the two properties enounced by Mayergoyz in the representation theorem [41]. This means that in order to be correctly described by the GPM, the system should obey to both wiping-out and congruency properties. The first property is essentially saying that the minor loops should be closed, and that after such a loop, the system should be back exactly in the same state (at the microscopic level) as in the initial state. The congruency refers to all the minor loops measured within the same limits for the independent variable (e.g., the magnetic field in ferromagnetic hysteresis). The systems correctly described by the GPM should have all the minor loops within the given limits of the independent variable congruent.

**Figure 3:**
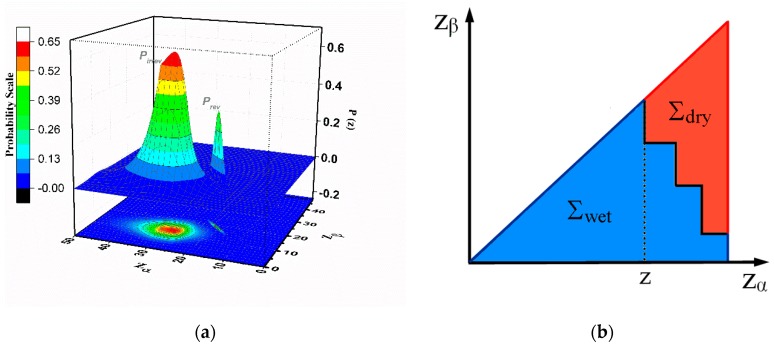
3D Preisach distribution in Generalized Preisach Model (**a**) and the Preisach plane separation line (**b**).

In the GPM the output value for the model is a sum between a value given by the irreversible component (as in CPM) and one given by the reversible part. In our case, if *z* is the independent variable (see Figure 2) and θ(z)  is the output for the system, we can define the reversible and irreversible Preisach distributions as $\;p_{rev}\left(z\right)$ and $p_{irrev}\left(z_{\alpha },z_{\beta }\right)$ (see Figure 3a). The value of the output is given by the sum of reversible $\left(\theta_{rev}\right)$ and irreversible $\left(\theta_{irrev}\right)$ components (on the total output only the hysterons in the “filled” state are accounted):

(1) $$\begin{array}{l} \theta \left(z\right)=\theta_{rev}\left(z\right)+\theta_{irrev}\left(z\right)\\ \theta_{rev}\left(z\right)=\int_{z_{\min}}^{z}p_{rev}\left(\zeta \right)d\zeta \\ \theta_{irrev}\left(z\right)=\iint_{\Sigma_{wet}}p_{irrev}\left(z_{\alpha },z_{\beta }\right)dz_{\alpha }dz_{\beta } \end{array}$$

**Figure 4:**
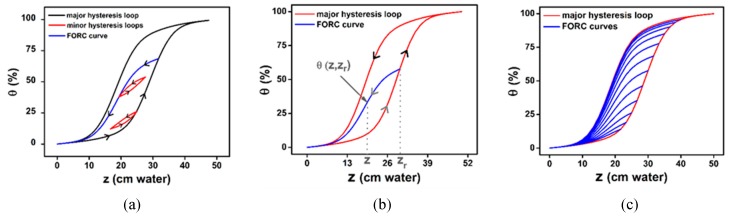
Simulation of the minor hysteresis loops when a small variation in the quantity of water occurs at one point on major hysteresis loop or First-Order Reversal Curve (FORC) (**a**); water content from soil $\left(\theta \left(z,{\;z}_{r}\right)\right)\;$ when the water level reached height *z* on FORC curve after the pore was filled with water up to the reversal point $z_{r}$ (**b**); and a set of FORC curves (**c**) simulating the drying curves starting from different points of wetting branch till the empty state.

In above equations, the z is the actual value of the independent variable (the height of the liquid measured from a selected reference point), $\left(z_{\min},z_{\max}\right)$ are the limits between the minimum and the maximum levels of the liquid, $\left(\Sigma_{wet},\Sigma_{dry}\right)$ are the regions in the Preisach plane (see Figure 3b) where the hysterons are on the upper or lower branches of their hysteresis loops, respectively. Essentially, the dependence on the history of the variations of the liquid level (given by the dependence on time of the independent variable *z*) are included in the irreversible component and is evidenced in the existence of the staircase line separating the hysterons in the “filled” or “empty” states. In Figure 4 we simulated few soil water characteristics curves, including minor loops and higher order curves obtained with analytical distributions for the following distributions for the Preisach distributions:

(2) $$p_{rev}\left(z\right)=\left(1-S\right)\frac{1}{\sqrt{2\pi }\sigma_{rev}}\exp\left[-\frac{{\left(z-z_{0r}\right)}^{2}}{2\sigma_{rev}^{2}}\right]$$

(3) $$\begin{array}{l} p_{irrev}\left(z_{\alpha },z_{\beta }\right)=S\frac{1}{2\pi \sigma_{\alpha }\sigma_{\beta }}\exp\left[-\frac{{\left(z_{\alpha }-z_{0\alpha }\right)}^{2}}{2\sigma_{\alpha }^{2}}\right]\\ \;\times \exp\left[-\frac{{\left(z_{\beta }-z_{0\beta }\right)}^{2}}{2\sigma_{\beta }^{2}}\right] \end{array}$$

where we have used Gaussian distributions for both components and assumed that the irreversible hysterons of pore suction have statistically independent switches from “filled” to “empty” state, respectively, a jump from “empty” to “filled”. The weight of the irreversible component is noted with *S* and represents the squareness of hysteresis loop; $\left(\sigma_{rev},\sigma_{\alpha },\sigma_{\beta }\right)$ are the standard deviations and $\left(z_{0r},z_{0\alpha },z_{0\beta }\right)\;$ the mean values of the three independent distributions taken into account in this case (the distribution for the reversible part and the distributions for the two switching values for *z*).

The major hysteresis loop is given by the wetting and drying branches of the hysteresis, seen as zero reversal curves, and minor hysteresis loops represent the wetting-drying cycles inside the Major Hysteresis Loop (MHL) (Figure 4a). The FORC curves are obtained as a set of primary scanning curves starting from the wetting branch of the MHL till the complete empty state (Figure 4b,c).

In soil, similar processes are happening very frequently when we consider alternating rainy and dry periods. In this case, simulations become an indispensable tool to characterize the unsaturated soil.

## 3. Identification Techniques Based on FORC Diagram Method

The most important problem in hysteresis modeling is related to the identification of the model parameters. If one starts with a hypothesis concerning the distributions, it is necessary to know analytical functions that describe them. In this case, the identification challenge can be reduced to the difficulty of finding a set of values for the parameters used in these functions. In our approach, we have to find a set of seven parameters $\left(S,\sigma_{rev},\;\sigma_{\alpha },\sigma_{\beta },z_{0r},z_{0\alpha },z_{0\beta }\right)$ in order to be able to use the function. It is a problem of optimization which can be approached using as a condition to obtain the best fit for a selected number of experimental curves. The selection of data is usually arbitrary and there is no warranty that the solution found is unique. Moreover, this method is not really efficient in many circumstances. This can be motivated by the fact that the functions selected in the model may not be realistic enough for the actual system which is analyzed or the assumptions made in the model are too unrealistic.

Consequently, in many circumstances, the parametric identification techniques are not useful and the so-called non-parametric identification techniques are preferred. The most successful non-parametric identification technique is the FORC method. It was initially designed by Mayergoyz [34] as an identification technique for the CPM applied for the ferromagnetic hysteresis. This method has the certain advantage of using well defined magnetic states and can be applied virtually to any hysteretic system descried by CPM. The method can be easily extended for the GPM and this is the reason we are giving a detailed presentation in this article.

In hysteresis process, the Major Hysteresis Loop (MHL) is considered as the zero-order curve and the primary scanning curve inside of them is called First-Order Reversal Curves (FORC), where the FORC have, by definition, the starting point ($z_{r}$ in Figure 4b) on the Major Hysteresis Loop. The MHL have two branches (ascendant and descendent) which means there are two sets of first-order reversal curves (re-wetting and re-drying curves) that can be measured, one for each branch of MHL. If we concentrate our discussion on the set of FORCs starting on the ascending branch of the MHL, we can specify that one such FORC starts on the major loop and ends at the empty state. The values of the output on a FORC (in our case θ) are depending on two independent variables: the input value in the reversal point, $z_{r}$, and the actual input value when θ is measured, *z*: $\theta_{FORC}=\theta \left(z,z_{r}\right)$, as in Figure 4b. If we compare this notation with the one made by Mayergoyz [34] in GPM, we can identify the pair $\left(z,z_{r}\right)$, with the mention that $\left(z_{\alpha },z_{\beta }\right)\;$ are the switching points of the FORCs measure starting on the ascending branch of MHL. In the identification technique for the CPM, but also for the GPM, it can be easily found the link between the Preisach distribution and the FORC data. The irreversible component of the Preisach distribution can be determined by using the second order mixed partial derivative with respect to the two switching points:

(4) $$p_{irrev}\left(z_{\alpha },z_{\beta }\right)=-\frac{\partial^{2}\theta_{FORC}\left(z_{\alpha },z_{\beta }\right)}{\partial z_{\alpha }\partial z_{\beta }}$$

However, this method should provide the Preisach distribution only for systems obeying the congruency and wiping-out properties. In practice, even in ferromagnetic hysteresis, it is virtually impossible to find perfect CPM/GMP systems. Most samples are showing a degree of disagreement especially with the congruency property. This is the main reason that for many years, the method was not actually used in practical applications. In 1999, a group from University of California, Davis, proposed a different approach. Pike and collaborators [39] have suggested the use of the Equation (4) to calculate an “experimental FORC distribution” directly from measured data and to use this distribution as a fingerprint for the magnetic samples. The representation of the experimental FORC distribution with contour plot lines is usually named FORC diagram.

Even if this new concept is taken into account, the FORC diagram technique still has a close relation with the Preisach model [42]. Most of the experimentalists using FORC method actually understand the FORC distribution as a slightly distorted version of the “real” Preisach distribution of the measured sample (showing the distribution of the hysteretic elements inside their samples as a function of the coercivity and interaction fields). Furthermore, in the last decade, an important effort was dedicated to the understanding of the physical significance of the experimental FORC diagrams for various hysteretic systems in the idea of transforming this method from a purely qualitative one to a powerful quantitative tool [43,44,45]. In magnetism, the method was used for a variety of samples with important applications in various areas starting from nanomagnetism [46], to geologic [47] and to archaeological samples [48]. In the same time, the use of this method was extended from magnetic materials to other hysteretic processes, like the ferroelectric hysteresis [49], or like the temperature, light-induced and pressure hysteresis in spin-transition materials [50,51,52,53]. In each case, the value of the FORC diagram technique was really evidenced only when an efficient physical model for the concrete case was developed and used in connection with the FORC method. For example, recent advances were made in the understanding of the quantitative relation between the experimental FORC diagrams and the physical entities in very simple nanostructured materials like in the 2D transversal and longitudinal magnetic nanowire samples [44,54,55].

So far, we have discussed only about the irreversible component of the hysteretic process; even though we know that reversible processes are always present in connection with the irreversible ones and that sometimes the weight of the reversible component could be quite high. Consequently, in order to accurately describe observed hysteretic phenomena, it is necessary to include as an indispensable ingredient the reversible part. However, it should be noted that this is not a simple task for any hysteresis model and that in many cases, the poor handling of this component is at the origin of great problems for the implemented models.

The reversible component was introduced traditionally in GPM by the use of zero coercivity hysterons (which are perfectly reversible) and their distribution can be determined directly from the susceptibility of the FORCs in the reversal points [40]. However, many studies made on magnetic systems, based on the first and second-order reversal curves, have shown that the reversible component is state dependent and coupled with the irreversible component (e.g., by mean field interactions) which, in these cases, are requiring more sophisticated methods to include correctly the reversible component in the determination of the total moment of the sample [56,57,58,59,60]. Taking into account these aspects, we have identified the physical significance of all parameters from capillary hysteresis in order to be able to use the FORC technique for real situations.

## 4. Results of FORC Identification

**Figure 5:**
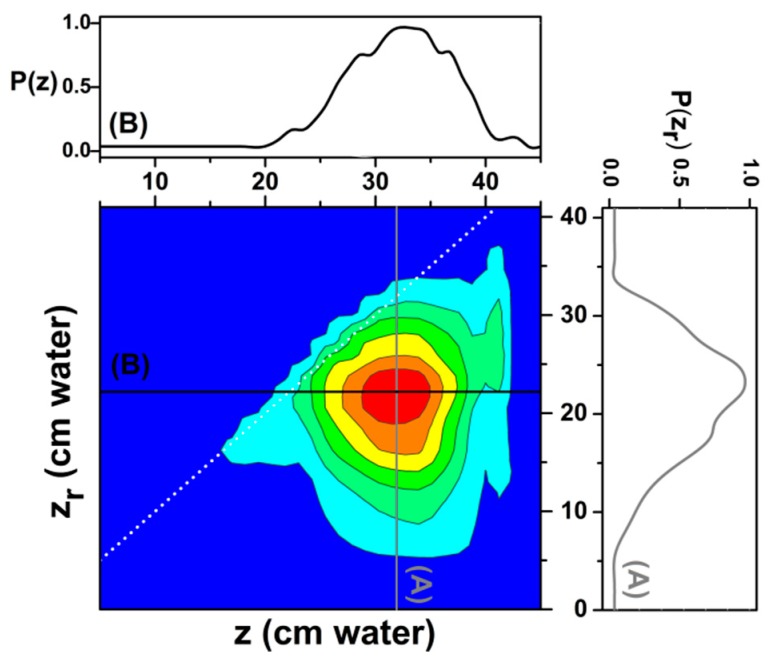
The distributions in FORC diagram have been performed for experimental data measured by Morrow and Harris [61]. Distribution ${P(z}_{r})$ was identified for the section of reversal points (line A) and P(z) for the section of current water pore level (line B).

In the present case, we have decided to identify both reversible and irreversible components from a well-known set of experimental data published by Morrow and Harris in 1965 [61], which has been used as input data by many other researchers since. In Figure 5, we show the FORC diagram with both reversible and irreversible components, as obtained from the mentioned set of experimental data using a customary numerical technique, developed by Pike and collaborators [39].

**Figure 6:**
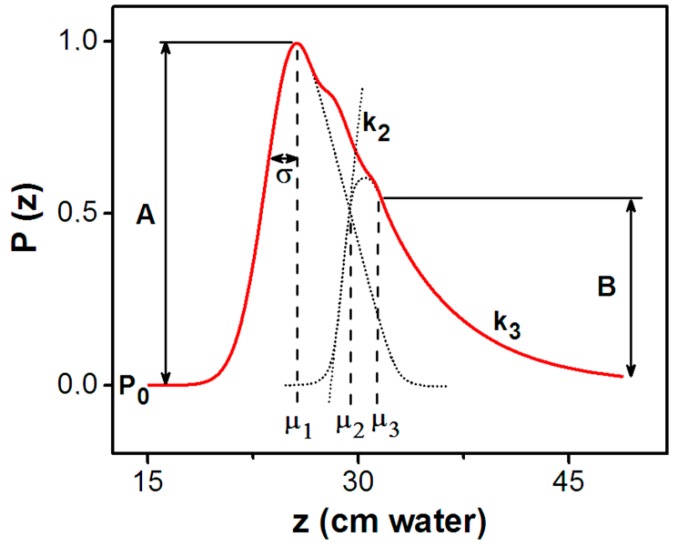
Chesler-Cram function identified from experimental FORC diagram.

Supplementary information can be obtained from the irreversible FORC distribution by providing two perpendicular sections (A and B lines), as observed in Figure 5. Taking into account the observed asymmetry on these sections we have chosen a Chesler-Cram peak function [62] for a fit (Figure 6). The Chesler-Cram function is a convolution of a Gaussian, exponential and hyperbolic tangent functions:

(5) $$\begin{array}{l} P(x)=P_{0}+A\left\{\exp\left[-\frac{{\left(x-\mu_{1}\right)}^{2}}{2\sigma }\right]+B\left[1-\frac{1-\tanh\left(k_{2}(x-\mu_{2})\right)}{2}\right]\times \exp\left[-k_{3}\frac{\left|x-\mu_{3}\right|+(x-\mu_{3})}{2}\right]\right\}\\ P_{irrev}(z,z_{r})=P(z)\cdot P(z_{r}) \end{array}$$

and depends on a number of nine parameters: $P_{0},\;A,\;B,\;\mu_{1},\;\mu_{2},\mu_{3},\sigma ,k_{2},k_{3}$.

The reversible component was fitted with a Gaussian Equation (6):

(6) $$P_{rev}(z)=P_{0\_rev}+A_{rev}\exp\left[-\frac{{\left(z-\mu_{rev}\right)}^{2}}{2\sigma_{rev}^{2}}\right]$$

The parameters of irreversible and reversible distributions for which the best fit were obtained are presented in Table 1.

Certainly, the major question that can be asked at this point is about the relation between the FORC distributions obtained from the experimental data and the physical reality of the samples that are analyzed here. A first step could be to see if a GPM with the reversible and irreversible Preisach distributions found from the fitting process of the experimental FORC distributions can provide a good approximation for the experimental data used in the identification process. If the answer is positive, the GPM could be an efficient tool for calculating the filling parameter for this pore system after any sequence of variations of the input parameter, which is the level of the liquid. In this way, a model with a strong predictive power can be developed and this can give a solid motivation for this type of identification technique.

We used the adapted Generalized Preisach Model in order to simulate the experimental data of drying FORC curves for soil samples. The Preisach distributions are formed by irreversible behavior of water in pores described by Equation (5) and the reversible pore water variation included in the model as Equation (6).

**Figure 7:**
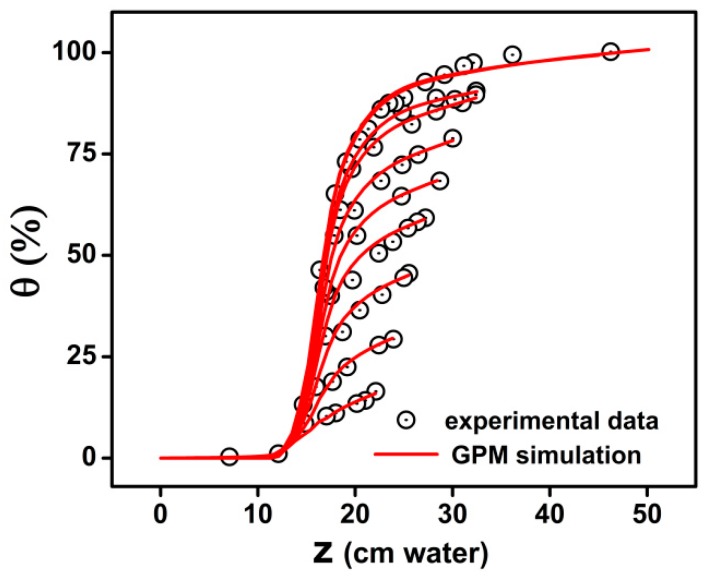
Drying FORC curves simulation by Generalized Preisach Model.

The reversible processes have proven to be an effective method that significantly increases the ability of the model to fit the experimental curves (Figure 7). In the figure we show the simulated curves using GPM and the experimental data.

An important point in the Preisach modeling is that in the absence of the reversible component, the non-zero initial slope observed in most published data concerning experimental first scanning curves cannot be reproduced correctly. The Classical Preisach Model without reversible component will always give a zero starting slope of these reversal curves.

## 5. Conclusions

We have calculated, from a set of experimental data from literature, the reversible and irreversible FORC distributions for the hysteretic capillary retention of liquid by soil. The experimental FORC distributions have been used as input in a Generalized Preisach Model with appropriate fitting functions. The results show the importance of the correct inclusion of the reversible component in the evaluation of the total results of simulation. The Preisach model with this architecture could reproduce with a high degree of accuracy the experimental data using a unique FORC distribution found from experimental data. This fact is suggesting that this procedure can be a practical method to use experimental data to generate a model able to provide significant prediction ability. From this point, additional techniques could be used to extend the method for other similar systems and for hysteretic processes in which kinetic effects could become important. In a further article, we shall provide quantitative comparison between our model and various other hysteresis models used in describing soil-moisture hysteresis in order to supplement and clarify the advantages and the limits of this approach.

## Figures and Tables

**Table 1:** The parameters value of irreversible and reversible distributions used in our simulation were.

-	*S*	*P*_0_	*A*	*σ*	*μ*_1_	*k*_2_	*μ*_2_	*B*	*k*_3_	*μ*_3_
*P*(*z_r_*)	0.65	0.0088	0.6625	29.0005	22.8078	0.2093	10.1015	0.4121	0.3054	26.6118
*P*(*z*)	0.65	0.0557	1.3109	35.5867	33.7578	0.0772	33.4031	−0.6025	0.2467	40.8232
-	(1−*S*)	*P*_0_*rev*_	*A*_rev_	*σ*_rev_	*μ*_rev_	-	-	-	-	-
*P_rev_*(*z*)	0.35	0.0219	0.3621	2.3923	26.0625	-	-	-	-	-
